# Adverse Events of COVID-19 Vaccination among the Saudi Population: A Systematic Review and Meta-Analysis

**DOI:** 10.3390/vaccines10122089

**Published:** 2022-12-07

**Authors:** Abdulaziz Alhossan, Amjad Khalid Alsaran, Afnan Hussain Almahmudi, Ziad Saad Aljohani, Mohammed Rajeh Albishi, Ahoud Khashman Almutairi

**Affiliations:** 1 Department of Clinical Pharmacy, College of Pharmacy, King Saud University, Riyadh P.O. Box 11451, Saudi Arabia; 2 College of Pharmacy, King Saud University, Riyadh P.O. Box 11451, Saudi Arabia; 3 Faculty of Pharmacy, Umm Al Qura University, Makkah P.O. Box 21955, Saudi Arabia; 4 Faculty of Pharmacy, University of Hail, Hail P.O. Box 2440, Saudi Arabia; 5 Ministry of National Guard Health Affairs (MNGHA), Riyadh P.O. Box 22490, Saudi Arabia; 6 College of Pharmacy, Qassim University, Unaizah-Qassim P.O. Box 6699, Saudi Arabia

**Keywords:** COVID-19, vaccine, adverse events, AstraZeneca, Pfizer-BioNTech

## Abstract

This systematic review and meta-analysis aimed to synthesize the evidence on the adverse events (AEs) of coronavirus disease 2019 (COVID-19) vaccinations in Saudi Arabia. A computerized search in MEDLINE via PubMed and OVID, Scopus, CENTRAL, and Web of Science was conducted using relevant keywords. The NIH tools were used for the quality assessment. A total of 14 studies (16 reports) were included. The pooled analysis showed that the incidence of AEs post-COVID-19 vaccination was 40.4% (95% CI:6.4% to 87%). Compared to the AstraZeneca vaccine, the Pfizer-BioNTech vaccine was associated with a lower risk ratio (RR) of wheezing (RR = 0.04), fever (RR = 0.32), chills (RR = 0.41), headache (RR = 0.47), dizziness (RR = 0.49), and joint pain (RR = 0.51). The Pfizer-BioNTech vaccine was associated with significantly higher RR of general allergic reactions (RR = 1.62), dyspnea (RR = 1.68), upper respiratory tract symptoms (RR = 1.71), and lymphadenopathy (RR = 8.32). The current evidence suggests that the incidence of AEs following COVID-19 vaccines is 40%; however, most of these AEs were mild and for a short time. The overall number of participants with AEs was higher in the Pfizer group compared to the AstraZeneca group; however, the AstraZeneca vaccine was associated with a higher RR of several AEs.

## 1. Introduction

Coronavirus disease 2019 (COVID-19) is an infectious disease that has resulted in the severe acute respiratory syndrome coronavirus 2 (SARS-CoV-2) [1]. COVID-19 has a lower fatality rate than the Middle East respiratory syndrome (MERS) but is spreading much faster [2,3]. To reduce the spread of SARS-CoV-2 and related deaths, several COVID-19 vaccines have been authorized for use in humans. These COVID-19 vaccines are based on several pharmacological methods: inactivated SARS-CoV-2 genes, an adenovirus vector, a protein subunit, and messenger RNA (mRNA) [4]. 

The mRNA-based vaccination (tozinameran or BNT162b2), publicly known as the Pfizer-BioNTech vaccine, emerged in 2020 during the COVID-19 pandemic. The mRNA vaccines act by directing human cells to generate spike protein, which is contained in SARS-CoV-2. The generated spike will protect the body against SARS-CoV-2 infection by stimulating the immune system [5,6]. The government of Saudi Arabia provides free vaccinations and online registration to all citizens and residents. The Ministry of Health has opened over 500 immunization clinics around the country to improve accessibility [7]. In December 2020, the Saudi Food and Drug Authority (SFDA) approved the tozinameran/BNT162b2 vaccine (Pfizer vaccine) [8]. On the other hand, mutated adenoviruses are used in a vector approach to prompt human cells to produce spike protein and the immune response to stimulate particular T cells for preventing COVID-19. 

In February 2021, the SFDA gave the green light to the distribution of ChAdOx1, a vector vaccine developed by a British-Swedish company with the help of Oxford University, known as the AstraZeneca vaccine [8,9]. In contrast to the mRNA and vector vaccines, the inactivated SARS-CoV-2 vaccine was generated by inactivating a specific strain of SARS-CoV-2 using -propiolactone, a chemical that may bind to virus genes and inhibit replication while leaving viral proteins intact. Inactivated viruses may be administered to boost the immune system without producing illness [10]. Recently, the SFDA has approved the use and distribution of Janssen (Johnson & Johnson) and Moderna vaccines; however, they have not yet authorized the use of any inactivated SARS-CoV-2 vaccine. Up to date, more than 66.7 million doses of vaccination have been received by the Saudi population, with 25.1 million individuals vaccinated, meaning 72% of the Saudi population.

Recently, active surveillance of the safety of COVID-19 vaccines (CoVaST) has been initiated; however, no results have been published yet [11]. There are a variety of published publications on the effects of COVID-19 vaccinations on human health, and each vaccine has its unique profile of risks and benefits. There was some inconsistency in these findings based on the specific vaccination and population studied. Due to differences in ethnic background and genetics, such heterogeneity is likely [12,13,14]. Symptoms or signs of upper respiratory inflammation, tachycardia, headache, shivering, myalgia, weakness throughout the body, and pain at the injection site were all documented as adverse drug reactions (ADRs) of the vaccinations [15,16]. This systematic review and meta-analysis aimed to synthesize the evidence on the adverse events (AEs) of COVID-19 vaccinations in Saudi Arabia.

## 2. Methods

We have followed the guidelines of the Preferred Reporting Items for Systematic Reviews and Meta-Analyses (PRISMA) checklist and Cochrane handbook for systematic reviews of interventions in reporting this study [17,18].

### 2.1. Eligibility Criteria

The studies were included based on the following criteria: 

Population: studies that were conducted on the Saudi population who received the COVID-19 vaccine.

Exposure: studies that included data about the Pfizer-BioNTech vaccine and/or the AstraZeneca vaccine.

Outcomes: studies documenting the AEs of one of the aforementioned vaccines or both. 

Study design: observational studies (case-control, cohort, and cross-sectional).

We excluded case reports, conference abstracts, and non-English studies.

### 2.2. Information Sources and Search Strategy

On April 20, 2022, we searched the following databases: MEDLINE via PubMed and OVID, Scopus, CENTRAL, and Web of Science, using the relevant keywords, such as (“Ad26COVS1”[Mesh] OR “COVID-19 Vaccines”[Mesh] OR “ChAdOx1 nCoV-19”[Mesh] OR “2019-nCoV Vaccine mRNA-1273”[Mesh] OR “BNT162 Vaccine”[Mesh]) AND (“Saudi Arabia”[Mesh] OR Kingdom of Saudi Arabia) to identify the relevant citations. Supplementary File S1 shows the detailed search term for each database. These databases were searched from inception to the date of search. Moreover, the reference lists of all included citations were searched. The retrieved citations were imported to EndNote X9 software, and duplicates were removed.

### 2.3. Selection Process

Using Microsoft Excel software, a screening sheet was created. the study ID, publication year, title, abstract, keywords, DOI, and URL were all included. The selection process was undertaken using a two-step screening technique by three independent reviewers (M.R.A, A.Y.A, and S.F.A). Step one was screening the title and abstract of all studies found via the literature search to determine which studies might proceed to step two (Full-text screening), where reviewers would read and assess whether each research met eligibility criteria. Any disagreement between the reviewers was solved by the judgment of the study supervisor (A.A). 

### 2.4. Data Items and Collection Process

Four independent reviewers extracted the following data from the included studies to an offline pre-prepared Excel sheet: demographic data of the included participants (age, gender, and residency), study characteristics (studies groups, study duration, total sample size, country, and main findings), and outcomes (incidence rate of adverse events following COVID-19 vaccination). 

### 2.5. Risk of Bias and Quality Assessment 

Using the National Institutes of Health (NIH) quality assessment tool for observational cohort, case-control, and cross-sectional studies, two authors (S.M.A and N.I.A) independently evaluated the risk of bias and the quality of each included article. Reviewers can critically evaluate the internal validity of research using this tool. The tool consists of 14 questions; studies that scored <7 were deemed as “poor”, from 7–9 were deemed as “fair”, and >9 were deemed as “Good”. A third author (A.A) resolved disagreements when the authors disagreed on a rating. A detailed list of these questions can be found in Supplementary File S2.

### 2.6. Data Synthesis

The prevalence of adverse events was calculated using the random-effects model with a 95% CI. Using the I^2^ statistic, we calculated the percentage of heterogeneity and inconsistency between studies, with values of 25%, 50%, and 75% deemed low, moderate, and high, respectively. The random-effect model was employed if the heterogeneity was considerable and I^2^ > 50%; otherwise, the fixed-effect model was utilized. The comprehensive meta-analysis was used for all statistical analyses (CMA; USA: version 3.3.070). To resolve heterogeneity, a sensitivity analysis was performed by removing one study in each scenario, known as a sequential sensitivity analysis. Furthermore, a subgroup analysis was performed to minimize the risk of inconsistency. To assess the difference between Pfizer-BioNTech and AstraZeneca in terms of AEs, we used the Review Manager 5.4 software to calculate the risk ratio (RR) between both groups using the Mantel–Haenszel model. Publication bias was assessed based on the criteria of Egger’s test, and a funnel plot was generated for the forest plots that included 10 studies or more.

## 3. Results

### 3.1. Study Selection

**Figure 1:**
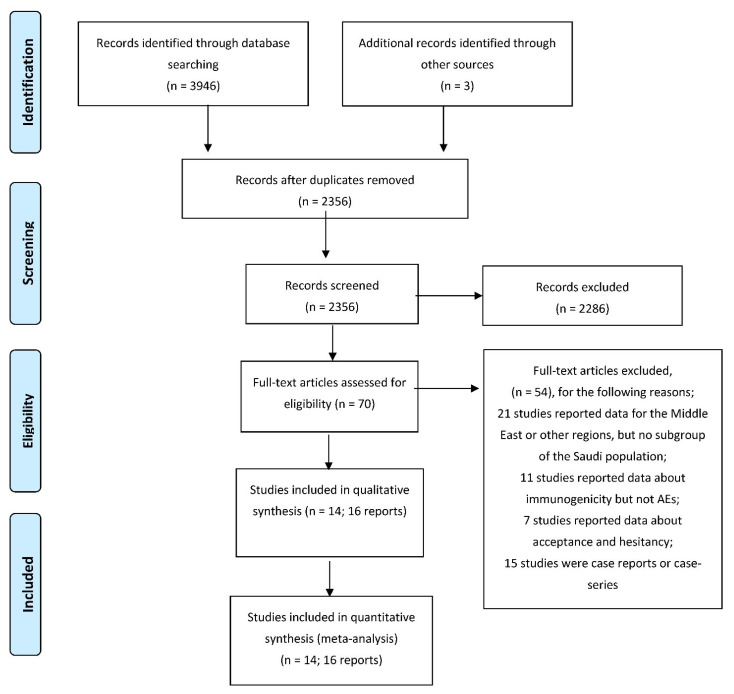
PRISMA flow diagram.

Based on our literature search, we found a total of 3949 relevant citations. After removing duplication, 2356 articles underwent title/abstract screening. Then, 2286 studies were deemed ineligible to our criteria. The full-text screening was performed on 70 articles, and only 14 studies (16 reports) were included in the qualitative (systematic review) and quantitative synthesis (meta-analysis) [7,19,20,21,22,23,24,25,26,27,28,29,30,31,32,33]. Figure 1 shows the PRISMA flow diagram of included studies.

### 3.2. Characteristics of Included Studies and Participants

All the included studies were cross-sectional except for one retrospective cohort study. Eight studies collected their data using an online survey, two used a telephone-based survey, two used traditional self-reported questionnaires, one used an interview questionnaire, and one used hospital databases for ADRs. Seven studies compared the Pfizer-BioNTech vaccine and AstraZeneca; five reported data for only Pfizer-BioNTech; and two reported data for only AstraZeneca. Both adults and children were included. More than half of the participants (52.17%) are males across the included studies. Alghamdi A. et al. conducted a study of 528 participants and published three reports; the first report compared males and females [27], the second report compared participants older or younger than 50 years [28], and the third report compared healthcare workers and non-healthcare workers [29]. Table 1 summarizes the baseline characteristics of included studies and participants. 

### 3.3. Quality of the Included Studies

Based on the NIH quality assessment tool for observational studies, six were deemed as “Good,” and eight were deemed as “Fair.” Supplementary File S2 shows the detailed quality assessment based on the NIH tool. 

### 3.4. Meta-Analysis

**Figure 2:**
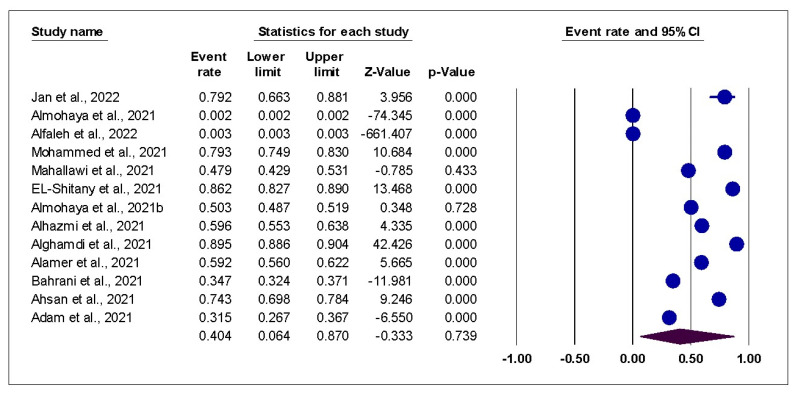
Forest Plot of overall AEs; shows the pooled analysis of overall AEs among vaccinated individuals.

**Figure 3:**
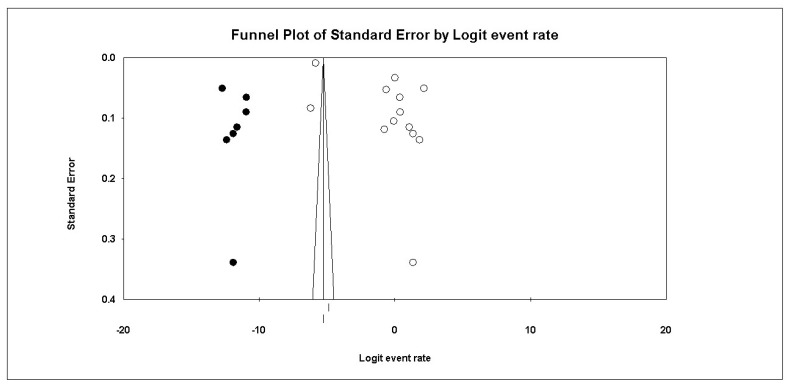
Funnel plot of standard error by the logit event rate.

Overall AEs: The pooled analysis of 13 studies showed that the incidence of AEs post-COVID-19 vaccination was 40.4% (95% CI:6.4% to 87%). The pooled data were heterogenous (I2 = 99%; *p* < 0.001), Figure 2 Sensitivity analysis could not solve the heterogeneity. Subgroup analysis showed that the incidence of AEs post-COVID-19 vaccination in studies that used online surveys was 62.5% (95% CI:52.1–72.0%), traditional questionnaires 13.6% (95% CI:0–99.7%), and other methods 13.7% (95% CI:0.2–92.8%). Based on the type of vaccine, the incidence of AEs post-COVID-19 vaccination in both vaccines was 43.7% (95% CI:1.9–96.9%), Pfizer-BioNTech vaccine 37.0% (95% CI:5.1–86.4%), AstraZeneca vaccine 34.7% (95% CI:32.4–37.1%). The funnel plot and Egger’s test showed a significant risk of publication bias (*p* = 0.002) which was solved by trimming three studies, resulting in a much smaller effect size of 26.1% (95% CI:5.0–70.2%), as shown in Figure 3.

**Figure 4:**
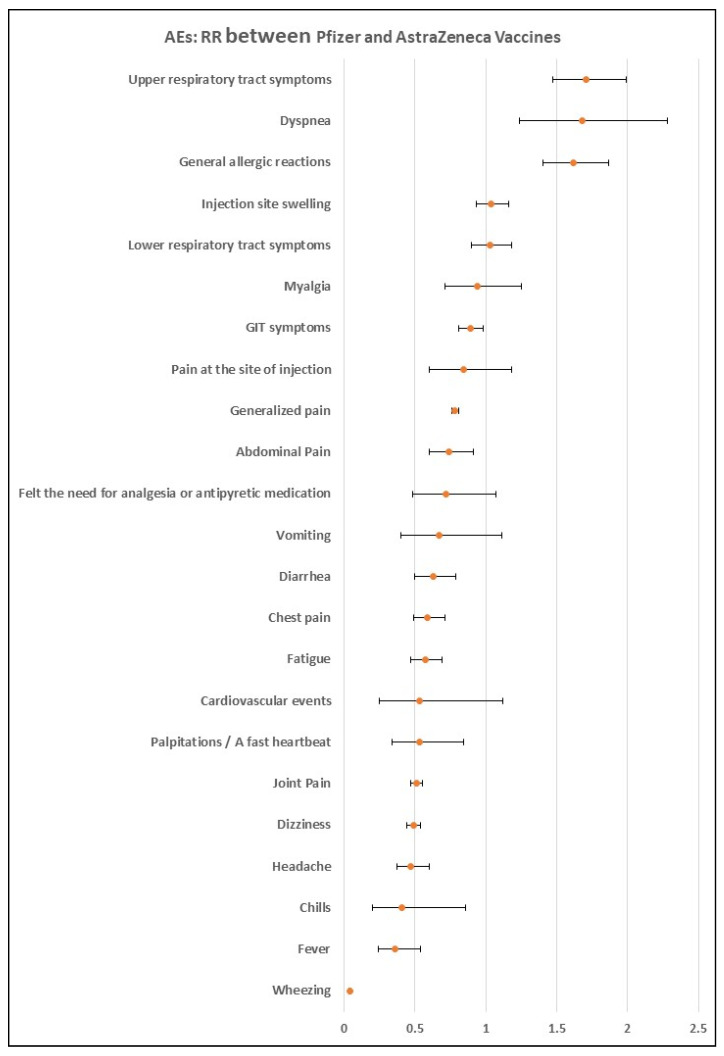
Forest plot of AEs in Pfizer group compared with AstraZeneca group.

Comparison between Pfizer-BioNTech and AstraZeneca vaccines: The pooled analysis showed that the Pfizer-BioNTech vaccine was associated with lower RR of several AEs, including fever (RR = 0.32, 95% CI: 0.30–0.35; I^2^ = 0%, *p* = 0.64), chills (RR = 0.41, 95% CI: 0.20–0.86; I^2^ = 99%; *p* < 0.001), headache (RR = 0.47, 95% CI: 0.37–0.60; I^2^ = 93%, *p* < 0.001), dizziness (RR = 0.49, 95% CI: 0.44–0.54; I^2^: 0%, *p* = 0.73), palpitations (RR = 0.53, 95% CI: 0.34–0.84; I^2^ = 83%; *p* = 0.02), fatigue (RR = 0.57, 95% CI: 0.47–0.69; I^2^ = 58%, *p* = 0.12), and diarrhea (RR = 0.63, 95% CI: 0.50–0.79; I^2^ = 0%, *p* = 0.52). On the other hand, the Pfizer-BioNTech vaccine was associated with significantly higher RR compared to the AstraZeneca vaccine in the following AEs: general allergic reactions (RR = 1.62, 95% CI: 1.40–1.87; I^2^ = NA), dyspnea (RR = 1.68, 95% CI: 1.24–2.28; I^2^ = 0%; *p* = 0.51), upper respiratory tract symptoms (RR = 1.71, 95% CI: 1.47–1.99; I^2^ = NA), and lymphadenopathy (RR = 8.32, 95% CI: 6.16–11.22; I^2^ = NA), as shown in Figure 4.

Most common AEs: Table 2 showed that the most common AEs in participants who received the Pfizer-BioNTech vaccine were pain at the site of injection 59.3% (95% CI:44.3–73.0%), myalgia 35.3% (95% CI:28.3–43.0%), fatigue 28.3% (95% CI:20.3–38.0%), joint pain 27.3% (95% CI:25.3–28.0%), and headache 25.3% (95% CI:22.3–29.0%). On the other hand, the most common AEs in participants who received the AstraZeneca vaccine were myalgia 70.7% (95% CI:67.0–80.8%), fatigue 60.8% (95% CI:31.0–80.8%), fever 57.7% (95% CI:42.0–80.8%), joint pain 55.5% (95% CI:53.0–80.8%), and pain at the site of injection 54.8% (95% CI:26.0–80.8%), as shown in Table S1.

The severity of AEs: Eight studies reported data on the severity of AEs [7,19,21,24,25,26,30,32]. Among the studies that reported data on both Pfizer and AstraZeneca vaccines [7,25,32], 12.06% had serious AEs, and 71.88% had non-serious AEs (RR = 0.17, 95% CI: 0.09–0.34; *p* < 0.0001). Regarding the participants who received the Pfizer vaccine only [19,21,24,26,30], 17.91% had serious AEs, and 78.73% had non-serious AEs (RR = 0.20, 95% CI: 0.14–0.30; *p* < 0.00001). One study reported data on participants who received the AstraZeneca vaccine [26]; 2.49% of them had serious AEs, and 76.35% had non-serious AEs (RR = 0.03, 95% CI: 0.02 –0.04; *p* < 0.00001), as shown in Table 3**.**

Dose-dependent AEs: Five studies reported data regarding the AEs experienced after the first dose and second dose of the Pfizer vaccine [21,23,24,26,30]. The pooled analysis showed that the first dose of the Pfizer vaccine was associated with significantly lower risks of chills (RR = 0.41, 95% CI: 0.36–0.48; *p* < 0.0001), fever (RR = 0.42, 95% CI: 0.31–0.55; *p* < 0.00001), headache (RR = 0.74, 95% CI: 0.66–0.83; *p* < 0.00001), joint pain (RR = 0.39, 95% CI: 0.24–0.61; *p* < 0.0001), lymph node swelling (RR = 0.48, 95% CI: 0.34–0.67; *p* < 0.0001), nausea and vomiting (RR = 0.72, 95% CI: 0.60–0.86; *p* = 0.0004), nerve inflammation symptoms (RR = 0.68, 95% CI: 0.55–0.84; *p* = 0.0004), tiredness (RR = 0.58, 95% CI: 0.40–0.84; *p* = 0.004), flu-like symptoms (RR = 0.36, 95% CI: 0.30–0.43; *p* < 0.0001), and body ache (RR = 0.60, 95% CI: 0.55–0.66; *p* < 0.0001), as shown in Table 4.

The interval between COVID-19 vaccination and AEs onset: Alfaleh et al. [20] reported that the median time of AEs onset post-Pfizer vaccine was significantly longer than AstraZeneca vaccine [1.96 (0.1–94.1) vs. 1.64 (0.1–33.4) days; *p* = 0.001). Similarly, Mohammed et al. [21] found that the median duration of AEs onset after the Pfizer vaccine was 2 days with an IQR of 11 days. They also showed that most events occurred within 24 h (72.6%), and 23.3% occurred between 24–48 h. Alhazmi et al. [25] reported that 84% of the participants experienced AEs within 24 h, 15% within 48 h, and 1% within 72 h, regardless of the type of vaccine. Likewise, Alamer et al. [30] showed that 72% of the participants who received the Pfizer vaccine experienced AEs within 24 h, 20% within 48 h, and 8% within 72 h. Ahsan et al. [32] mentioned that among participants who received either AstraZeneca or Pfizer vaccines, 87.1% experienced AEs within 24 h and 12.9% between 24–48 h. When comparing males vs. females, Alghamdi et al. [27] demonstrated that males were associated with delayed onset of AEs (15 ± 14 h vs. 12.4 ± 10.5 h; *p* = 0.037). They also compared participants older and younger than 50 years, and their findings showed that 65.4% of the participants younger than 50 experienced AEs within 24 h, compared to 63% in the elderly group [28]. When they compared healthcare workers vs. the general population, they found that the onset of AEs was significantly delayed in the healthcare workers group (16 ± 15.4 vs 12.2 ± 10.2 h; *p* = 0.002) [29].

## 4. Discussion

In this systematic review and meta-analysis, our findings showed a high rate of overall AEs among the included studies. This rate was very high in the online surveys compared to the traditional self-reported or telephone-based questionnaires, which is expected as online surveys are associated with a higher response rate [34]. The overall AEs associated with the Pfizer-BioNTech vaccine were slightly higher than the AstraZeneca vaccine. However, the Pfizer-BioNTech vaccine was associated with lower RR of several AEs, including wheezing, fever, chills, headache, dizziness, joint pain, palpitations, fatigue, chest pain, diarrhea, abdominal pain, generalized pain, and GIT symptoms. On the other hand, the Pfizer-BioNTech vaccine was associated with significantly higher RR than the AstraZeneca vaccine in the following AEs: general allergic reactions, dyspnea, upper respiratory tract symptoms, and lymphadenopathy. This is consistent with earlier research showing that Pfizer-BioNTech vaccines had fewer adverse effects than Oxford-AstraZeneca and other companies [35,36]. On the other hand, Klugar et al. reported that the prevalence of local AEs was higher in mRNA-based vaccines compared to viral vector-based vaccines (78.3% vs. 70.4%); however, the difference was not statistically significant (*p* = 0.064). In terms of systemic AEs, the prevalence was significantly higher in the viral vector-based vaccine (87.2% vs. 61%; *p* < 0.001) [37]. Previously published findings of phase III clinical studies and vaccine fact sheets consistently reported post-vaccination effects for individuals who got the second dosage [23,38,39,40,41]. Menni et al. recently conducted a prospective observational study and revealed results consistent with previous studies [42]. Similar to our findings, a recent systematic review and meta-analysis showed that headache, fatigue, and fever were the most reported systemic AEs. Most local AEs recorded were pain, swelling, and redness at the injection site [43].

Local and systemic AEs are examples of post-immunization complications [44]. The best vaccines could protect against a targeted virus without causing undesired side effects. However, in real life, all vaccines have the risk of inducing some AEs that may or may not be related to the immunization itself. In most cases, the cause of adverse responses after vaccination is unclear and may be attributable to the vaccine’s adjuvant, stabilizer, or preservative [43,45]. The majority of COVID-19 vaccine-related adverse effects are mild, consistent with the experience of earlier vaccinations. There may be a correlation between IgE-mediated responses and the vaccine’s active antigen, leading to local adverse reactions [46].

For most currently used and formerly used vaccinations, the incidence of vaccine-induced anaphylactic shock is around one in a million doses administered [47]. Vaccine-induced anaphylaxis was almost 10 times more common with Pfizer-BioNTech and Moderna than earlier vaccinations [45]. Unfortunately, the cause of this negative response is yet to be determined. Some inactive components or results of the vaccine manufacturing process, such as lipid or the polyethylene glycol (PEG)-lipid component of the vaccine, may increase susceptibility to non-IgE-related mast-cell activation or complement activation in some individuals [45]. When delivered in vivo, mRNA vaccines are protected from degradation via a lipid-based nanoparticle carrier technology. PEG 2000 lipid conjugate, which provides a hydrophilic layer, significantly stabilizes this carrier system and extends the half-life [47,48]. The vaccines developed by Pfizer-BioNTech and Moderna are the first mRNA vaccines to be authorized. Accordingly, the mechanism of allergic responses to mRNA vaccines remains unknown. The mechanism of allergic reactions to mRNA vaccines was unknown until the Pfizer-BioNTech vaccine, and the Moderna vaccine was granted Emergency Use Authorization (EUA).

Recent systematic reviews and meta-analyses have been published that assess the effectiveness and safety of COVID-19 vaccinations in light of the existing evidence. The most prevalent AEs were fatigue and headache, which is consistent with our findings [49]. Other meta-analyses reported that myalgia, fever, fatigue, and headache were the most common systemic AEs following injection, whereas swelling, erythema, and pain at the injection site were the most prevalent local AEs [50,51,52]. The number of participants who had adverse events and reactions after receiving an injection of an RNA-based vaccination has been found to be greater than with other vaccine types [50]. Serious AEs after immunization against COVID-19 were not documented in any investigations. Notably, the results of this investigation and the existing literature imply that AEs may be moderate and transient owing to the initiation of active immune responses. Since immunization has been shown to be very effective in lowering the likelihood of hospitalization, intensive care unit admission, mechanical ventilation, and mortality, it is recommended that it be continued and the occurrence of these adverse events be anticipated [48,50,51,52]. Current recommendations from the US CDC and the Saudi Ministry of Health (MOH) for the medical management of post-vaccination AEs emphasize the use of symptomatic therapy. 

This is the first systematic review and meta-analysis of AEs associated with COVID-19 vaccinations in Saudi Arabia. This study covered more than 13% of the Saudi population and around 20% of all vaccinated individuals in Saudi Arabia. However, our study has some limitations, including the high heterogeneity, which could not be solved by sensitivity or subgroup analysis. Moreover, the observed publication bias is another limitation. Due to the scarcity of data, we could not compare the vaccinated and non-vaccinated and early and late AEs.

In conclusion, the current evidence suggests that the incidence of AEs following COVID-19 vaccines is 40%; however, most of these AEs were mild and lasted for a short time. The most common systemic AEs were myalgia, fatigue, headache, dizziness, fever, and vomiting, whereas the most common local AEs were pain and swelling at the site of injection and joint pain. The overall number of participants with AEs was higher in the Pfizer group compared to the AstraZeneca group; however, the AstraZeneca vaccine was associated with a higher RR of several AEs. Since immunization may be anticipated to be very effective in lowering the likelihood of hospitalization, intensive care unit admission, mechanical ventilation, and mortality, it is recommended that it be continued and the occurrence of these adverse events be anticipated.

## Figures and Tables

**Table 1:** Summary of Included studies.

ID	Study Design	Method of Data Collection	Study Duration	Sample Size	Vaccines	Age (Years) **	Inclusion Criteria	Male
Jan et al., 2022	cross-sectional	Questionnaire	May 10 to 20, 2021	147	Pfizer and AstraZeneca	32.2 (19–49)	All participants with Sickle Cell Disease who received Pfizer or AstraZeneca vaccines	79 (53.7%)
Almohaya et al., 2021	retrospective cohort	Hospital Databases	February 1st to March 31st, 2021	71221	Pfizer	32 (16–109)	Participants experienced AEs following the Pfizer vaccine	39884 (56)
Alfaleh et al., 2022	cross-sectional	Questionnaire	December 2020 to March 2021 *	4432572	Pfizer and AstraZeneca	16 to ≥65	Individuals had at least one dose of any batch of any of the Pfizer or AstraZeneca vaccines	NA
Mohammed et al., 2021	cross-sectional	Online survey	February to March 2021	386	Pfizer	18 to ≥65	Any individual who took the Pfizer vaccine in Saudi Arabia	177 (45.9)
Mahallawi et al., 2021	cross-sectional	Online survey	February 1st to June 30th, 2021 *	365	Pfizer and AstraZeneca	45.1 ± 14.7	Participants who received both doses of either the Pfizer or AstraZeneca COVID-19 vaccine	306 (83.8)
EL-Shitany et al.,2021	cross-sectional	Online survey	January 10 to 21, 2021	455	Pfizer	16 to ≥65	Individuals who received at least one dose of the Pfizer COVID-19 vaccine.	163 (35.8%)
Almohaya et al., 2021b	cross-sectional	Online survey	June 1 to 8, 2021	3639	Pfizer	18 to ≥65	A resident of the KSA at the time of enrollment and have received at least one dose of the Pfizer vaccine	1337 (36.7)
Alhazmi et al., 2021	cross-sectional	Online survey	April 7 to April 28, 2021	515	Pfizer and AstraZeneca	26 ± 9	Any individual took Pfizer or AstraZeneca vaccines	221 (43%)
Alghamdi N. et al., 2021	cross-sectional	Questionnaire	-	4170	Pfizer and AstraZeneca	16 to ≥65	Any individual took Pfizer or AstraZeneca vaccines in Saudi Arabia	1296 (31.08)
Alghamdi A. et al., 2021	cross-sectional	Telephone-based survey	February 28 to March 12, 2021	528	AstraZeneca	16 to ≥65	Individuals who received the AstraZeneca COVID-19 vaccine	263 (49.81)
Alghamdi A. et al., 2021b	cross-sectional	Telephone-based survey	February 28 to March 12, 2021	528	AstraZeneca	16 to ≥65	Individuals who received the AstraZeneca COVID-19 vaccine	263 (49.81)
Alghamdi A. et al., 2021c	cross-sectional	Telephone-based survey	February 28 to March 12, 2021	528	AstraZeneca	16 to ≥65	Individuals who received the AstraZeneca COVID-19 vaccine	263 (49.81)
Alamer et al., 2021	cross-sectional	Online survey	1st August to 24th August of 2021	965	Pfizer	16 ± 2	Children received single or double doses of the Pfizer vaccine	460 (48)
Bahrani et al., 2021	cross-sectional	Telephone-based survey	April to May 2021	1592	AstraZeneca	37.4 ± 9.6	Individuals who received the first dose of the AstraZeneca vaccine	1290 (81)
Ahsan et al., 2021	cross-sectional	Online survey	March 30 to May 13, 2021)	397	Pfizer and AstraZeneca	34.43 ± 6.73	Individuals who received at least one dose of either the Pfizer or AstraZeneca vaccine	209 (52.6)
Adam et al., 2021	cross-sectional	Online survey	March to May 2021	330	Pfizer and AstraZeneca	18 to ≥65	Participants who received either one or two doses of the AstraZeneca or Pfizer vaccine	216 (65.5)

* The duration of vaccine administration. ** Age presented as mean ± SD, median (IQR), or range.

**Table 2:** Most common local and systemic AEs in both vaccines.

AEs	Pfizer	AstraZeneca
Myalgia	35.3% (28.3–43.0%)	70.7% (67.0–80.8%)
Fatigue	28.3% (20.3–38.0%)	60.8% (31.0–80.8%)
Fever	18.3% (14.3–23.0%)	57.7% (42.0–80.8%)
Joint Pain	27.3% (25.3–28.0%)	55.5% (53.0–80.8%)
Pain at the site of injection	59.3% (44.3–73.0%)	54.8% (26.0–80.8%)
Headache	25.3% (22.3–29.0%)	45.5% (36.0–80.8%)
Chills	10.3% (6.3–14.0%)	40.6% (21.0–80.8%)
Generalized pain	7.3% (0.3–41.0%)	26.9% (01.0–80.8%)
Injection site swelling	12.3% (7.3–19.0%)	25.2% (23.0–80.8%)
Dizziness	20.3% (11.3–33.0%)	24.6% (04.0–80.8%)
Vomiting	4.3% (1.3–12.0%)	18.4% (14.9–22.6%)
Chest pain	8.3% (5.3–13.0%)	13.1% (11.0–80.8%)
GIT symptoms	8.3% (1.3–28.0%)	10.1% (06.0–80.8%)
Diarrhea	5.3% (3.3–06.0%)	9.6% (8.2–11.1%)
Abdominal Pain	7.3% (6.3–7.0%)	09.1% (08.0–80.8%)
Numbness	6.3% (3.3–11.0%)	8.5% (6.4–11.2%)
Nausea	7.3% (3.3–14.0%)	07.2% (02.0–80.8%)
Palpitations/A fast heartbeat	6.3% (5.3–7.0%)	4.9% (0.7–26.1%)
Dyspnea	6.3% (4.3–11.0%)	3.7% (2.9–4.6%)
Sore throat	1.3% (0.3–15.0%)	0.9% (0–16.9%)
Lymphadenopathy	2.3% (1.3–6.0%)	0.6% (0.5–0.8%)
Hospitalization due to side effects	8.3% (6.3–11.0%)	0.0% (0–8%)
High Blood pressure	1.3% (0.3–13.0%)	NA

**Table 3:** Severity of AEs.

Vaccines	Number of Studies	Serious vs. Non-Serious (%)	RR (95% CI)	Heterogeneity
AstraZeneca and Pfizer	3	12.07 vs. 71.88	(RR = 0.17, 95% CI: 0.09–0.34; *p* < 0.0001)	I^2^ = 89%, *p* < 0.001
Pfizer	5	17.91 vs. 78.73	(RR = 0.20, 95% CI: 0.14–0.30; *p* < 0.00001)	I^2^ = 98%, *p* < 0.001
AstraZeneca	1	2.49 vs. 76.35	(RR = 0.03, 95% CI: 0.02–0.04; *p* < 0.00001)	-

**Table 4:** Dose-dependent AEs.

AEs	Number of Studies	RR (95% CI)	Heterogeneity
Bad rash all over the body	2	(RR = 0.88, 95% CI: 0.36–2.14; *p* = 0.78)	I^2^ = 11%; *p* = 0.29
Chest pain	2	(RR = 0.90, 95% CI: 0.71–1.14; *p* = 0.38)	I^2^ = 0%; *p* = 0.37
Chills	4	(RR = 0.41, 95% CI: 0.36–0.48; *p* < 0.0001)	I^2^ = 0%; *p* = 0.45
Diarrhea	2	(RR = 1.25, 95% CI: 0.48–3.29; *p* = 0.65)	I^2^ = 32%; *p* = 0.22
Difficulty of Breathing	3	(RR = 0.97, 95% CI: 0.75–1.26; *p* = 0.82)	I^2^ = 23%; *p* = 0.27
Dizziness and giddiness	2	(RR = 0.87, 95% CI: 0.75–1.01; *p* = 0.07)	I^2^ = 0%; *p* = 0.83
Elevated blood pressure	2	(RR = 0.89, 95% CI: 0.38–2.08; *p* = 0.78)	I^2^ = 11%; *p* = 0.29
Fatigue	4	(RR = 0.73, 95% CI: 0.51–1.04; *p* = 0.08)	I^2^ = 93%; *p* < 0.0001
Fever	5	(RR = 0.42, 95% CI: 0.31–0.55; *p* < 0.00001)	I^2^ = 88%; *p* < 0.0001
Headache	5	(RR = 0.74, 95% CI: 0.66–0.83; *p* < 0.00001)	I^2^ = 48%; *p* = 0.11
Hypersensitivity Symptoms	2	(RR = 0.79, 95% CI: 0.45–1.40; *p* = 0.42)	I^2^ = 66%; *p* = 0.09
Injection site swelling and redness	3	(RR = 1.08, 95% CI: 0.94–1.25; *p* = 0.26)	I^2^ = 45%; *p* = 0.16
Joint pain	2	(RR = 0.39, 95% CI: 0.24–0.61; *p* < 0.0001)	I^2^ = 50%; *p* = 0.13
Lips swelling	2	(RR = 1.24, 95% CI: 0.70–2.18; *p* = 0.46)	I^2^ = 0%; *p* = 0.59
Lymph node swelling	4	(RR = 0.48, 95% CI: 0.34–0.67; *p* < 0.0001)	I^2^ = 29%; *p* = 0.24
Nausea and vomiting	3	(RR = 0.72, 95% CI: 0.60–0.86; *p* = 0.0004)	I^2^ = 0%; *p* = 0.48
Nerve Inflammation Symptoms	2	(RR = 0.68, 95% CI: 0.55–0.84; *p* = 0.0004)	I^2^ = 43%; *p* = 0.19
Pain at the site of the injection	5	(RR = 1.02, 95% CI: 0.93–1.12; *p* = 0.67)	I^2^ = 83%; *p* = 0.0001
Sore throat	2	(RR = 1.00, 95% CI: 0.81–1.24; *p* = 0.98)	I^2^ = 0%; *p* = 0.53
Tiredness	2	(RR = 0.58, 95% CI: 0.40–0.84; *p* = 0.004)	I^2^ = 45%; *p* = 0.18
Flu-like symptoms	2	(RR = 0.36, 95% CI: 0.30–0.43; *p* < 0.0001)	I^2^ = 0%; *p* = 0.84
Body ache	2	(RR = 0.60, 95% CI: 0.55–0.66; *p* < 0.0001)	I^2^ = 74%; *p* = 0.05

## Data Availability

All data are presented within the study text or its Supplementary Files.

## Supplementary Materials

The following supporting information can be downloaded at: https://www.mdpi.com/article/10.3390/vaccines10122089/s1, File S1: Used Keywords for search term, File S2: NIH tool of quality assessment, Table S1: AEs among all vaccinated patients, Pfizer vaccine, and AstraZeneca vaccine.
