# Supplementary material for: Adverse Events of COVID-19 Vaccination among the Saudi Population: A Systematic Review and Meta-Analysis
Source: Vaccines (Basel). 2022 Dec 7;10(12):2089. doi: 10.3390/vaccines10122089 (PMC9783010; doi:10.3390/vaccines10122089)
Supplement: Supplementary file 1 [file vaccines-10-02089-s001.zip › Supplementary file S1.pdf]

Supplementary file S1: Used Keywords for search term

Database	Search term	Results
PubMed	(((((Ad26.COV2.S) OR (Ad26COVS1)) OR (JNJ-78436735)) OR (COVID-19 Vaccine Janssen)) OR (Johnson & Johnson vaccine)) AND (((((Saudi Arabia) OR (Kingdom of Saudi Arabia)) OR (Saudisc Arabea)) OR (saudis arabia)) OR (Saudi Arab)) AND (((Spikevax) OR (mRNA-1273)) OR (Moderna vaccine)) AND (((((Saudi Arabia) OR (Kingdom of Saudi Arabia)) OR (Saudisc Arabea)) OR (saudis arabia)) OR (Saudi Arab)) AND (((((Vaxzevria) OR (AZD1222)) OR (ChAdOx1 nCoV-19)) OR (AstraZeneca vaccine)) OR (Oxford/AstraZeneca vaccine)) AND (((((Saudi Arabia) OR (Kingdom of Saudi Arabia)) OR (Saudisc Arabea)) OR (saudis arabia)) OR (Saudi Arab)) AND (((((((Comirnaty) OR (Tozinameran)) OR (BNT162b2)) OR (BioNTech/Pfizer vaccine)) OR (Pfizer vaccine)) OR (BioNTech vaccine)) OR (mRNABNT162b2)) AND (((((Saudi Arabia) OR (Kingdom of Saudi Arabia)) OR (Saudisc Arabea)) OR (saudis arabia)) OR (Saudi Arab)) AND (COVID19 Vaccines OR COVID19 Vaccine OR SARS-CoV-2 Vaccines OR SARS CoV 2 Vaccine OR Vaccine, SARS-CoV-2 OR SARS2 Vaccines OR SARS2 Vaccine OR Coronavirus Disease 2019 Vaccines OR Coronavirus Disease 2019 Vaccine OR Coronavirus Disease 19 Vaccine OR COVID 19 Vaccine OR 2019 nCoV Vaccine OR SARS Coronavirus 2 Vaccines) AND (Saudi Arabia OR Kingdom of Saudi Arabia OR saudis arabia OR Saudi Arab)	741
Scopus	ALL((Ad26.COV2.S OR Ad26COVS1 OR JNJ-78436735 OR "COVID-19 Vaccine Janssen" OR "Johnson and Johnson vaccine") AND ("Saudi Arabia" OR "Kingdom of Saudi Arabia" OR "saudis arabia" OR "Saudi Arab")) AND ALL((Spikevax OR mRNA-1273 OR "Moderna vaccine") AND ("Saudi Arabia" OR "Kingdom of Saudi Arabia" OR "saudis arabia" OR "Saudi Arab")) AND ALL((Vaxzevria OR AZD1222 OR ChAdOx1 nCoV-19 OR "AstraZeneca vaccine" OR "Oxford/AstraZeneca vaccine" OR "AstraZeneca/Oxford vaccine" OR "Oxford vaccine") AND ("Saudi Arabia" OR "Kingdom of Saudi Arabia" OR "saudis arabia" OR "Saudi Arab")) AND ALL((Comirnaty OR Tozinameran OR BNT162b2 OR "BioNTech/Pfizer vaccine" OR "Pfizer vaccine" OR "BioNTech vaccine" OR mRNABNT162b2) AND ("Saudi Arabia" OR "Kingdom of Saudi Arabia" OR "saudis arabia" OR "Saudi Arab")) AND ALL(("COVID19 Vaccines" OR "COVID19 Vaccine" OR "SARS-CoV-2 Vaccines" OR "SARS CoV 2 Vaccine" OR "Vaccine, SARS-CoV-2" OR "SARS2 Vaccines" OR "SARS2 Vaccine" OR	2525

	"Coronavirus Disease 2019 Vaccines" OR "Coronavirus Disease 2019 Vaccine" OR "Coronavirus Disease 19 Vaccine" OR "COVID 19 Vaccine" OR "2019 nCoV Vaccine" OR "SARS Coronavirus 2 Vaccines") AND ("Saudi Arabia" OR "Kingdom of Saudi Arabia" OR "saudis arabia" OR "Saudi Arab"))	
Web of Science	(((((Ad26.COV2.S) OR (Ad26COVS1)) OR (JNJ-78436735)) OR (COVID-19 Vaccine Janssen)) OR (Johnson & Johnson vaccine)) AND (((((Saudi Arabia) OR (Kingdom of Saudi Arabia)) OR (Saudisc Arabea)) OR (saudis arabia)) OR (Saudi Arab)) AND (((Spikevax) OR (mRNA-1273)) OR (Moderna vaccine)) AND (((((Saudi Arabia) OR (Kingdom of Saudi Arabia)) OR (Saudisc Arabea)) OR (saudis arabia)) OR (Saudi Arab)) AND (((((Vaxzevria) OR (AZD1222)) OR (ChAdOx1 nCoV-19)) OR (AstraZeneca vaccine)) OR (Oxford/AstraZeneca vaccine)) AND (((((Saudi Arabia) OR (Kingdom of Saudi Arabia)) OR (Saudisc Arabea)) OR (saudis arabia)) OR (Saudi Arab)) AND (((((((Comirnaty) OR (Tozinameran)) OR (BNT162b2)) OR (BioNTech/Pfizer vaccine)) OR (Pfizer vaccine)) OR (BioNTech vaccine)) OR (mRNABNT162b2)) AND (((((Saudi Arabia) OR (Kingdom of Saudi Arabia)) OR (Saudisc Arabea)) OR (saudis arabia)) OR (Saudi Arab)) AND (COVID19 Vaccines OR COVID19 Vaccine OR SARS-CoV-2 Vaccines OR SARS CoV 2 Vaccine OR Vaccine, SARS-CoV-2 OR SARS2 Vaccines OR SARS2 Vaccine OR Coronavirus Disease 2019 Vaccines OR Coronavirus Disease 2019 Vaccine OR Coronavirus Disease 19 Vaccine OR COVID 19 Vaccine OR 2019 nCoV Vaccine OR SARS Coronavirus 2 Vaccines) AND (Saudi Arabia OR Kingdom of Saudi Arabia OR saudis arabia OR Saudi Arab)	976
Medline via Ovid	(((((Ad26.COV2.S) OR (Ad26COVS1)) OR (JNJ-78436735)) OR (COVID-19 Vaccine Janssen)) OR (Johnson & Johnson vaccine)) AND (((((Saudi Arabia) OR (Kingdom of Saudi Arabia)) OR (Saudisc Arabea)) OR (saudis arabia)) OR (Saudi Arab)) AND (((Spikevax) OR (mRNA-1273)) OR (Moderna vaccine)) AND (((((Saudi Arabia) OR (Kingdom of Saudi Arabia)) OR (Saudisc Arabea)) OR (saudis arabia)) OR (Saudi Arab)) AND (((((Vaxzevria) OR (AZD1222)) OR (ChAdOx1 nCoV-19)) OR (AstraZeneca vaccine)) OR (Oxford/AstraZeneca vaccine)) AND (((((Saudi Arabia) OR (Kingdom of Saudi Arabia)) OR (Saudisc Arabea)) OR (saudis arabia)) OR (Saudi Arab)) AND (((((((Comirnaty) OR (Tozinameran)) OR (BNT162b2)) OR (BioNTech/Pfizer vaccine)) OR (Pfizer vaccine)) OR (BioNTech vaccine)) OR (mRNABNT162b2)) AND (((((Saudi Arabia) OR (Kingdom of Saudi Arabia)) OR (Saudisc Arabea)) OR (saudis arabia)) OR (Saudi Arab))	190

	AND (COVID19 Vaccines OR COVID19 Vaccine OR SARS-CoV-2 Vaccines OR SARS CoV 2 Vaccine OR Vaccine, SARS-CoV-2 OR SARS2 Vaccines OR SARS2 Vaccine OR Coronavirus Disease 2019 Vaccines OR Coronavirus Disease 2019 Vaccine OR Coronavirus Disease 19 Vaccine OR COVID 19 Vaccine OR 2019 nCoV Vaccine OR SARS Coronavirus 2 Vaccines) AND (Saudi Arabia OR Kingdom of Saudi Arabia OR saudis arabia OR Saudi Arab)	
CENTRAL	(((((Ad26.COV2.S) OR (Ad26COVS1)) OR (JNJ-78436735)) OR (COVID-19 Vaccine Janssen)) OR (Johnson & Johnson vaccine)) AND (((((Saudi Arabia) OR (Kingdom of Saudi Arabia)) OR (Saudisc Arabea)) OR (saudis arabia)) OR (Saudi Arab)) AND (((Spikevax) OR (mRNA-1273)) OR (Moderna vaccine)) AND (((((Saudi Arabia) OR (Kingdom of Saudi Arabia)) OR (Saudisc Arabea)) OR (saudis arabia)) OR (Saudi Arab)) AND (((((Vaxzevria) OR (AZD1222)) OR (ChAdOx1 nCoV-19)) OR (AstraZeneca vaccine)) OR (Oxford/AstraZeneca vaccine)) AND (((((Saudi Arabia) OR (Kingdom of Saudi Arabia)) OR (Saudisc Arabea)) OR (saudis arabia)) OR (Saudi Arab)) AND (((((((Comirnaty) OR (Tozinameran)) OR (BNT162b2)) OR (BioNTech/Pfizer vaccine)) OR (Pfizer vaccine)) OR (BioNTech vaccine)) OR (mRNABNT162b2)) AND (((((Saudi Arabia) OR (Kingdom of Saudi Arabia)) OR (Saudisc Arabea)) OR (saudis arabia)) OR (Saudi Arab)) AND (COVID19 Vaccines OR COVID19 Vaccine OR SARS-CoV-2 Vaccines OR SARS CoV 2 Vaccine OR Vaccine, SARS-CoV-2 OR SARS2 Vaccines OR SARS2 Vaccine OR Coronavirus Disease 2019 Vaccines OR Coronavirus Disease 2019 Vaccine OR Coronavirus Disease 19 Vaccine OR COVID 19 Vaccine OR 2019 nCoV Vaccine OR SARS Coronavirus 2 Vaccines) AND (Saudi Arabia OR Kingdom of Saudi Arabia OR saudis arabia OR Saudi Arab)	11
