# Supplementary material for: Adverse Events of COVID-19 Vaccination among the Saudi Population: A Systematic Review and Meta-Analysis
Source: Vaccines (Basel). 2022 Dec 7;10(12):2089. doi: 10.3390/vaccines10122089 (PMC9783010; doi:10.3390/vaccines10122089)
Supplement: Supplementary file 1 [file vaccines-10-02089-s001.zip › Supplementary Table S1.pdf]

Supplementary Table S1: AEs among all vaccinated patients, Pfizer vaccine, and AstraZeneca vaccine

AEs			Prevalence/RR (95% CI)	Studies	Heterogeneity
Overall AEs	Pooled data	All Vaccinated	0.404 (0.064 – 0.870)	13	99%; <0.001
	Data collection	Online survey	0.625 (0.521 – 0.720)	8	98%; <0.001
		Questionnaire	0.136 (0.0 - 0.997)	2	99%; <0.001
		Others	0.137 (0.002 – 0.928)	3	99%; <0.001
	Vaccine	Both	0.437 (0.019 – 0.969)	7	99%; <0.001
		Pfizer	0.370 (0.051 – 0.864)	5	99%; <0.001
		AstraZeneca	0.347 (0.324 – 0.371)	1	NA
Fever		All Vaccinated	0.342 (0.264 – 0.430)	6	95%; <0.001
		Pfizer	0.183 (0.143 – 0.230)	9	95.05%; <0.001
		AstraZeneca	0.572 (0.427 – 0.706)	8	99.30%; <0.001
		Pfizer vs. AstraZeneca (RR)	0.36 [0.24, 0.54]	6	98%; p<0.001
Headache		All Vaccinated	0.339 (0.270 – 0.415)	5	93.28; <0.001
		Pfizer	0.259 (0.228 – 0.293)	11	92.37; <0.001
		AstraZeneca	0.454 (0.369 – 0.541)	7	97.36; <0.001
		Pfizer vs. AstraZeneca (RR)	0.47 (0.37 - 0.60)	6	93%; <0.001
GIT symptoms		Pfizer	0.080 (0.018 – 0.287)	2	97.45%; <0.001
		AstraZeneca	0.100 (0.061 – 0.159)	4	97.89%; <0.001
		Pfizer vs. AstraZeneca (RR)	0.89 (0.81 - 0.98)	1	NA
Diarrhea		All Vaccinated	0.054 (0.017 – 0.161)	2	49.7%; 0.158
		Pfizer	0.051 (0.037 – 0.068)	4	78.67%; 0.003
		AstraZeneca	0.096 (0.082 – 0.111)	2	0%; 0.462
		Pfizer vs. AstraZeneca (RR)	0.63 (0.50 - 0.79)	2	0%; 0.52
Dizziness		All Vaccinated	0.081 (0.058 – 0.113)	2	0%; 0.904
		Pfizer	0.201 (0.114 – 0.330)	5	98.35%; <0.001
		AstraZeneca	0.240 (0.047 – 0.666)	2	84.69%; 0.011
		Pfizer vs. AstraZeneca (RR)	0.49 (0.44 - 0.54)	2	0%; 0.73
Chest pain		All Vaccinated	0.099 (0.090 – 0.108)	1	NA
		Pfizer	0.083 (0.050 – 0.134)	3	91.13%; <0.001
		AstraZeneca	0.133 (0.117 – 0.151)	1	NA
		Pfizer vs. AstraZeneca (RR)	0.59 (0.49 – 0.71)	1	NA
Dyspnea		All Vaccinated	0.036 (0.023 – 0.055)	4	60%; 0.057
		Pfizer	0.069 (0.040 – 0.117)	4	93%; <0.001
		AstraZeneca	0.037 (0.029 – 0.046)	3	0%; 0.56
		Pfizer vs. AstraZeneca (RR)	1.68 (1.24 – 2.28)	2	0%; 0.51
Generalized pain		All Vaccinated	0.019 (0.003 – 0.124)	1	NA
		Pfizer	0.071 (0.008 – 0.413)	3	98%; <0.001
		AstraZeneca	0.260 (0.010 – 0.923)	2	91%; <0.001
		Pfizer vs. AstraZeneca (RR)	0.78 (0.76 – 0.81)	1	NA
Myalgia		All Vaccinated	0.187 (0.036 – 0.586)	2	98%; <0.001
		Pfizer	0.356 (0.286 – 0.432)	1	NA
		AstraZeneca	0.701 (0.678 – 0.723)	2	86%; <0.001
		Pfizer vs. AstraZeneca (RR)	0.94 (0.71 – 1.25)	1	NA
Joint Pain		All Vaccinated	0.110 (0.039 – 0.274)	4	98%; <0.001
		Pfizer	0.270 (0.254 – 0.286)	3	97%; <0.001
		AstraZeneca	0.556 (0.535 – 0.581)	1	NA

	Pfizer vs. AstraZeneca (RR)	0.51 (0.47 – 0.55)	1	NA
Abdominal Pain	All Vaccinated	0.079 (0.072 – 0.088)	1	NA
	Pfizer	0.070 (0.064 – 0.076)	3	0%; 0.425
	AstraZeneca	0.095 (0.081 – 0.110)	1	NA
	Pfizer vs. AstraZeneca (RR)	0.74 (0.60 – 0.91)	1	NA
Numbness	Pfizer	0.065 (0.036 – 0.116)	2	62%; 0.10
	AstraZeneca	0.085 (0.064 – 0.112)	1	NA
Vomiting	All Vaccinated	0.169 (0.139 – 0.204)	1	NA
	Pfizer	0.043 (0.014 – 0.120)	5	97%; <0.001
	AstraZeneca	0.184 (0.149 – 0.226)	1	NA
	Pfizer vs. AstraZeneca (RR)	0.67 (0.40 – 1.11)	1	NA
Palpitations / A fast heartbeat	All Vaccinated	0.111 (0.102 – 0.121)	1	NA
	Pfizer	0.065 (0.054 – 0.079)	4	63%; 0.041
	AstraZeneca	0.049 (0.007 – 0.261)	3	99%; <0.001
	Pfizer vs. AstraZeneca (RR)	0.53 (0.34 – 0.84)	2	83%; 0.02
Cardiovascular events	Pfizer vs. AstraZeneca (RR)	0.53 (0.25 – 1.12)	1	NA
Upper respiratory tract symptoms	Pfizer vs. AstraZeneca (RR)	1.71 (1.47 – 1.99)	1	NA
Lower respiratory tract symptoms	Pfizer vs. AstraZeneca (RR)	1.03 (0.90 – 1.18)	1	NA
Lymphadenopathy	Pfizer	0.025 (0.010 – 0.063)	3	87% <0.001
	AstraZeneca	0.006 (0.005 – 0.008)	2	77%; 0.033
	Pfizer vs. AstraZeneca (RR)	8.32 (6.16 - 11.22)	1	NA
Wheezing	Pfizer vs. AstraZeneca (RR)	0.040 (0.034 – 0.046)	2	56%; 0.129
Pain at the site of injection	All Vaccinated	0.448 (0.208 – 0.715)	6	99%; <0.001
	Pfizer	0.595 (0.442 – 0.731)	8	99%;<0.001
	AstraZeneca	0.548 (0.260 – 0.808)	5	99%; <0.001
	Pfizer vs. AstraZeneca (RR)	0.84 (0.60 – 1.18)	4	90%;<0.001
Injection site swelling	Pfizer	0.120 (0.070 – 0.199)	5	97%; <0.001
	AstraZeneca	0.250 (0.230 – 0.273)	1	NA
	Pfizer vs. AstraZeneca (RR)	1.04 (0.93 – 1.16)	1	NA
Hospitalization due to side effects	Pfizer	0.086 (0.065 – 0.112)	1	NA
	AstraZeneca	0 (0-0.005)	1	NA
Visited the emergency department post vaccination	Pfizer vs. AstraZeneca (RR)	0.68 (0.04 – 10.24)	1	NA
Felt the need for analgesia or antipyretic medication	Pfizer vs. AstraZeneca (RR)	0.72 (0.48 – 1.07)	1	NA
General allergic reactions	Pfizer vs. AstraZeneca (RR)	1.62 (1.40 – 1.87)	1	NA
High Blood pressure	Pfizer	0.010 (0.001 - 0.135)	2	99%; <0.001
	Pfizer vs. AstraZeneca (RR)	3.02 (0.14 – 62.80)	1	NA
Pain in testis	Pfizer vs. AstraZeneca (RR)	0.20 (0.01 – 4.93)	1	NA
Fatigue	All Vaccinated	0.516 (0.356 – 0.673)	3	97% <0.001
	Pfizer	0.287 (0.207 – 0.381)	7	98%%; <0.001
	AstraZeneca	0.601 (0.312 – 0.833	4	99%; <0.001
	Pfizer vs. AstraZeneca (RR)	0.57 (0.47 – 0.69)	2	58% 0.12
Nausea	All Vaccinated	0.057 (0.020 – 0.150)	4	93%%; <0.001
	Pfizer	0.073 (0.035 – 0.144)	7	94%; <0.001
	AstraZeneca	0.076 (0.020 – 0.256)	3	89%; <0.001
	Pfizer vs. AstraZeneca (RR)	0.29 (0.03 – 3.07)	2	66% 0.08
Chills	All Vaccinated	0.20 (0.158 – 0.249)	5	85%; <0.001

	Pfizer	0.102 (0.069 – 0.147)	6	91%; <0.001
	AstraZeneca	0.40 (0.21 – 0.625)	4	99%; <0.001
	Pfizer vs. AstraZeneca (RR)	0.41 (0.20 – 0.86)	3	99%; <0.001
Sore throat	All Vaccinated	0.017 (0.001 – 0.256)	2	98%; <0.001
	Pfizer	0.014 (0.001 – 0.152)	3	98%; <0.001
	AstraZeneca	0.009 (0.00 – 0.169)	2	96%; <0.001
	Pfizer vs. AstraZeneca (RR)	2.41 (0.68 – 8.54)	1	NA
